# Auditory Processing in Noise: A Preschool Biomarker for Literacy

**DOI:** 10.1371/journal.pbio.1002196

**Published:** 2015-07-14

**Authors:** Travis White-Schwoch, Kali Woodruff Carr, Elaine C. Thompson, Samira Anderson, Trent Nicol, Ann R. Bradlow, Steven G. Zecker, Nina Kraus

**Affiliations:** 1 Auditory Neuroscience Laboratory and Department of Communication Sciences, Northwestern University, Evanston, Illinois, United States of America; 2 Department of Speech and Hearing Sciences, University of Maryland, College Park, Maryland, United States of America; 3 Department of Linguistics, Northwestern University, Chicago, Illinois, United States of America; 4 Department of Neurobiology & Physiology, Northwestern University, Evanston, Illinois, United States of America; 5 Department of Otolaryngology, Northwestern University, Chicago, Illinois, United States of America; McGill University, CANADA

## Abstract

Learning to read is a fundamental developmental milestone, and achieving reading competency has lifelong consequences. Although literacy development proceeds smoothly for many children, a subset struggle with this learning process, creating a need to identify reliable biomarkers of a child’s future literacy that could facilitate early diagnosis and access to crucial early interventions. Neural markers of reading skills have been identified in school-aged children and adults; many pertain to the precision of information processing in noise, but it is unknown whether these markers are present in pre-reading children. Here, in a series of experiments in 112 children (ages 3–14 y), we show brain–behavior relationships between the integrity of the neural coding of speech in noise and phonology. We harness these findings into a predictive model of preliteracy, revealing that a 30-min neurophysiological assessment predicts performance on multiple pre-reading tests and, one year later, predicts preschoolers’ performance across multiple domains of emergent literacy. This same neural coding model predicts literacy and diagnosis of a learning disability in school-aged children. These findings offer new insight into the biological constraints on preliteracy during early childhood, suggesting that neural processing of consonants in noise is fundamental for language and reading development. Pragmatically, these findings open doors to early identification of children at risk for language learning problems; this early identification may in turn facilitate access to early interventions that could prevent a life spent struggling to read.

## Abstract 2

The neural coding of consonants in noise predicts early literacy skills in pre-reading children, providing a biological marker that may help to target early intervention.

## Author Summary

Learning to read is a chief developmental milestone with lifelong consequences; although there are effective interventions for struggling readers, an ongoing challenge has been to identify candidates for intervention at a young-enough age. We measured the precision of the neural coding of consonants in noise, and found that pre-reading children (4 y old) with stronger neural processing had superior early literacy skills; one year later they were also stronger emerging readers. We applied the same neural coding measure to a cohort of older children: in addition to predicting these children’s literacy achievement, we could reliably predict which of the children had received a diagnosis of a reading impairment. Taken together, these results suggest that the neural coding of speech in noise plays a fundamental role in language development. Children who struggle to listen in noisy environments may struggle to make meaning of the language they hear on a daily basis, which can in turn set them at risk for literacy challenges. Evaluating the neural coding of speech in noise may provide an objective neurophysiological marker for these at-risk children, opening a door to early and specific interventions that may stave off a life spent struggling to read.

## Introduction

Three aspects of auditory-neurophysiological processing have often been associated with literacy: variability of neural firing [1,2], auditory system timing [3,4], and processing detailed acoustic features such as those found in consonants [5,6]. This neural coding is thought to play a pivotal role in reading and language development [5,7,8] and may reflect the precision of neural processing in the central auditory system, which likely develops through the integrated neural coding of speech across multiple timescales, including prosodic, syllabic, and phonemic acoustic information [8–10]. Although children are provided access to these sonic fundamentals in their everyday lives, these experiences often occur in adverse listening environments (classrooms, outdoors, wailing siblings) in which children need to tune out competing sounds to tune into speech. Indeed, noise places stringent demands on sensory processing, and individuals with language-based learning problems often have perceptual deficits in noise across modalities [11–15]. Background noise limits access to redundant acoustic cues that are accessible to listeners in quiet. In principle, noise may obfuscate both the neural processing of an individual acoustic event (such as a phoneme) and the formation of consistent representations of successive events (such as words or sentences); see, for example, [16]. Should children with poor processing in noise grow up forced to make sense of speech in these noisy environments, they may fall behind their peers in language development.

Auditory system precision—especially the neural processing of speech in noise—is correlated to literacy; that is, struggling readers perform poorly on behavioral tests of auditory processing [4] and have reduced auditory response fidelity and impaired neural coding of rapid auditory stimuli compared to good readers [2, 17]. Therefore, these brain–behavior links likely reflect neural mechanisms underlying reading in general, as opposed to a parochial deficit in clinical populations. It remains open to debate, however, what role these neural mechanisms play developmentally with respect to reading, in part because it remains debated if auditory function is consistently implicated in reading impairment at all [18]. Alternate accounts for the origins of reading impairment include sluggish processing in the magnocellular pathway [19,20], multimodal perceptual deficits grounded in inefficient short-term memory [21], and poor processing in cortical “reading networks” that lead to auditory impairments [22]. There are likely many reasons that a child may be a poor reader, including genetic and environmental; while understanding the factors that cause reading impairment is an important goal, it is also important to predict which children will struggle when they begin to read. Thus, from a pragmatic standpoint our aim is to define a neurophysiological marker that might identify these children.

To date, auditory-neurophysiological markers of literacy have only been observed in children and adults who have received prolonged, formal instruction. But the process of learning to read itself may induce changes in substrate reading skills [23,24] and their neural foundations [25]. Further compounding the problem is the challenge of predicting future literacy skills. There have been promising experiments reporting differences between groups of children (e.g., an at-risk group versus a control group or a group of children who receive a diagnosis versus a group who does not). But substantial overlap between groups (resulting in modest effect sizes) tends to thwart clinically-meaningful predictions in individual children [26–28]. Early identification of children at risk for reading problems is crucial; interventions that are provided early enough can bring struggling pre-readers in line with their peers and offset years of reading difficulties [29,30]. For example, in a prospective study of language-impaired children, Bishop and Adams reported that literacy development proceeded smoothly in children whose oral language problems were resolved by age 5.5 y [31]. This motivates us to investigate early language skills, and their neural correlates, in preschoolers.

It has long been argued that reading skills are linked to the processing of rapid auditory information, meaning that struggling readers have particular problems with auditory temporal processing [4,5,32], including the perception and neural coding of dynamic speech elements [11,15]. Here, then, we evaluated neural processing of a consonant-vowel syllable in background noise. This processing in noise relies upon neural synchrony—that is, consistent and uniform neural population discharges [33]. In humans, neural synchrony in response to the crucial phonemic features of speech may be measured through the frequency following response (FFR, a scalp-recorded auditory evoked potential that is also known as the auditory brainstem response to complex sounds, or cABR). The neural circuitry important for language development may not engage faithfully during everyday listening experiences because of a breakdown in synchronous neural firing exacerbated by background noise. As a consequence of this poor online processing in noise, these children may lag behind their peers in language development. Previous studies in older children have established relationships between FFR properties and reading, and therefore provide empirical grounding for the current investigation [2,3,11]. We also evaluated children’s phonological skills because phonological processing—knowledge and manipulation of the sound structure of spoken language—is a chief pre-reading skill that is deficient in children with dyslexia [8]. Our hypothesis is that background noise disrupts brain mechanisms involved in literacy development; we therefore predict that children with poor auditory-neurophysiological responses to speech in noise exhibit poorer early literacy skills than their peers.

## Results

### Neural Coding of Consonants in Noise Predicts Phonological Processing (Experiment 1)

**Fig 1:**
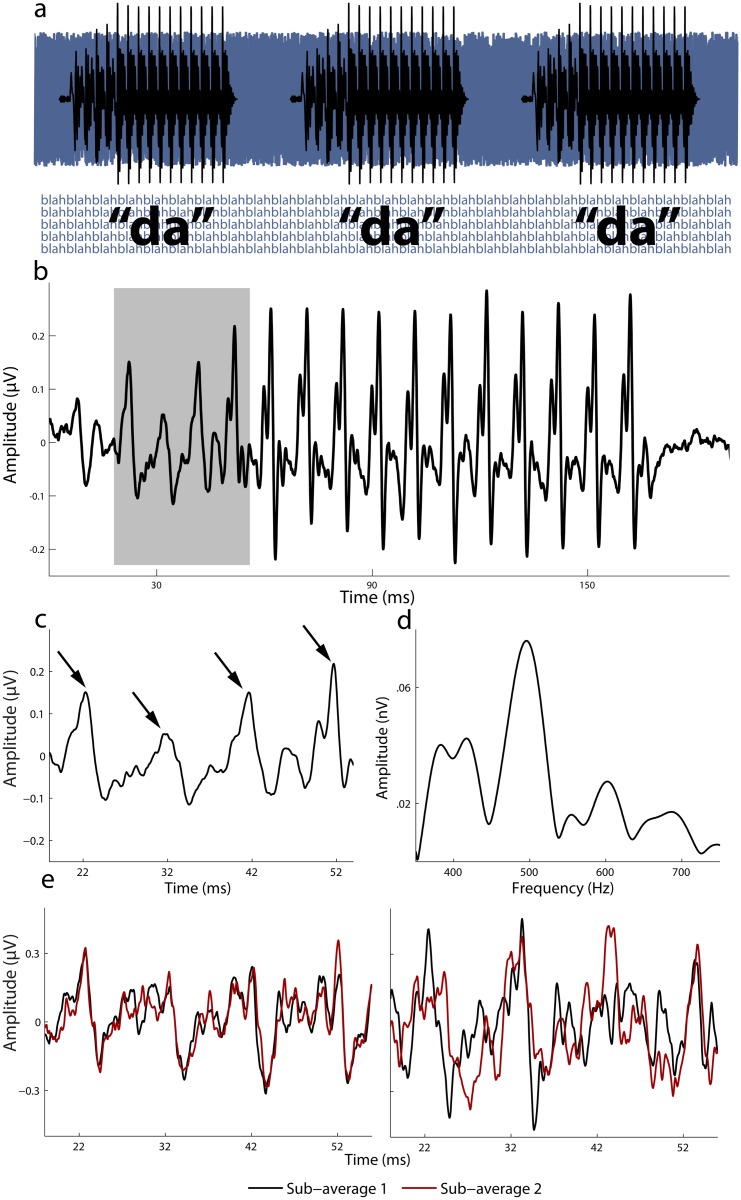
Overview of the auditory-neurophysiological biomarker and three derived neural measures. (A) Recording paradigm: [da] is presented repeatedly over a continuous background track of nonsense sentences spoken by multiple talkers. (B) A time-domain average waveform of the response. The response shows many of the physical characteristics of the eliciting stimulus. The gray box highlights the time region of the response that corresponds to the consonant transition (the region of interest). (C) The peaks of interest are identified here with arrows. (D) A frequency domain representation of the grand average response to the consonant transition. (E) To illustrate the trial-by-trial stability measure, two representative subjects are shown. One pair of sub-averages each is shown for a subject with high stability and one with poor stability (right).

We constructed a statistical model incorporating three aspects of the neural coding of consonants in noise: trial-by-trial stability [1,2], neural timing [3,15], and representation of spectral features that convey phonemic identity (see Fig 1) [3,11] in a cohort of 4-y-old children who had not yet learned to read (*n* = 37, 21 female; mean [M] age 54.41 months, standard deviation [SD] 3.56). These quantify different aspects of auditory processing and have all been linked to reading skills in older children. Although these metrics come from a single neurophysiological recording, they are not strongly intercorrelated within an individual (see S1 Fig); thus, each provides unique information about the coding of different linguistic and paralinguistic parameters.

**Fig 2:**
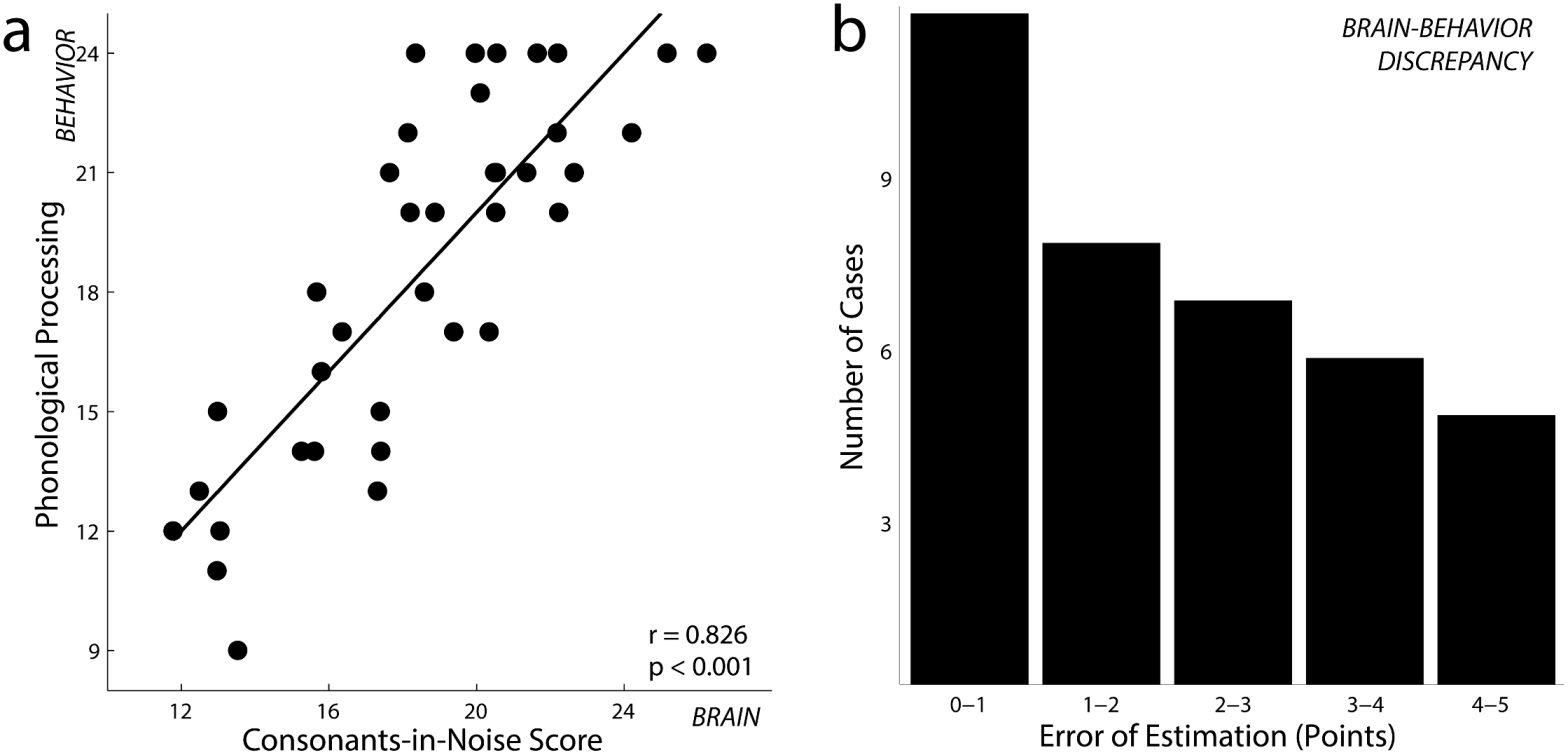
(A) In Year 1 (Experiment 1) each child’s score on the phonological processing test is plotted against the model’s predicted scores (*n* = 37). The two are highly correlated (*r* = 0.826, *p* < .001; when a correction is applied for the unreliability of the psychoeducational test, *r* = 0.870, *p* < .001). (B) A histogram of the error of estimation (the difference between a preschooler’s actual and predicted scores). For a majority of children, the model predicts scores within 2 points on the test. Please refer to the S1 Data for data underlying this figure.

We found that neural coding of consonants in noise strongly predicted phonological processing in pre-readers over and above demographic factors (CELF P-2 Phonological Awareness; Δ*R* ^2^ = 0.488, *F*[9,24] = 4.121, *p* = 0.003; total *R* ^2^ = 0.684, *F*[12,36] = 4.328, *p* = 0.001; see Table 1 and Fig 2A; when the correlation was adjusted for test-retest variability of the behavioral test, *R* ^2^ = 0.757; see also S1 Text for a cross-validation of this model). For the majority of children, our model predicted scores within 2 points on the test, which is less than a 10% margin of error (difference between actual scores and model-predicted scores; median = 1.97 points; range, 0.17–5.66 points; see Fig 2B). Our results suggest that the precision and stability of coding consonants in noise parallels emergent literacy skills across a broad spectrum of competencies—all before explicit reading instruction begins.

**Table 1:** Neural coding of consonants in noise predicts preschooler’s phonological processing. These model parameters are applied in Experiments 2–4.

Predictor	Δ*R* ^2^	*β*
Step 1	0.196^‡^	
Sex^a^		-0.076
Age		0.390*
Non-verbal IQ		0.114
Step 2	0.488	
Sex		-0.162
Age		0.452**
Non-verbal IQ		0.351*
*Neural timing*		
Peak 21		0.420**
Peak 31		-0.332‡
Peak 41		-0.055
Peak 51		-0.117
*Spectral features*		
H4		0.120
H5		-0.514**
H6		0.052
H7		0.300*
*Neural stability*		0.266
**Total R^2^**	0.848^‡^	

^a^Dummy-coded, males = 0, females = 1.

^‡^ *p* < 0.10

*p < 0.05

** *p* < 0.01

Statistical model predictions from this regression were used in subsequent analyses. The idea is that model predictions reflect a “consonants-in-noise score” that may be correlated to performance cross-sectionally and longitudinally on additional behavioral tests. For Experiments 2 through 4, we measured FFRs to consonants in noise, computed the same measures of neural coding in those children, and applied regression parameters from Experiment 1 to those children’s responses. This effectively predicts performance on this test of phonological processing even though, as detailed below, we did not conduct this particular test in all children. In no cases did we refit the data with new regression models.

### Neural Coding of Consonants in Noise Predicts Multiple Preliteracy Skills (Experiment 2)

Having constructed a model based on phonological processing, we explored whether model predictions generalized to multiple tests of preliteracy. We applied our predictive model from Experiment 1 to 20 3-y-olds (9 female; M = 43.35 months, SD 2.50) in whom we could not administer the test of phonological processing (see Methods) but could conduct neurophysiological testing. We used the model parameters estimated in Experiment 1 and combined these “consonants-in-noise scores” with those from the 37 children in that experiment. Neural coding of consonants in noise predicted performance on a test of rapid automatized naming, an additional key preliteracy skill that is thought to be highly predictive of future reading success across languages [34,35] (higher predicted scores correlated with faster naming; *r*[55] = -0.550, *p* < .001). Neural coding also predicted children’s memory for spoken sentences (*r*[55] = 0.516, *p* < .001), a test that combines auditory working memory with knowledge of grammar—an additional substrate skill that contributes to literacy development and is often deficient in children with dyslexia and/or language impairment [36].

We also split this cohort into the two age groups. Recall that the “consonants-in-noise score” was fit to the 37 4-y-olds from Experiment 1, and we applied these regression weights to the 20 3-y-olds in whom we could not measure phonological processing. In the 4-y-olds the “consonants-in-noise” score predicted memory for spoken sentences (*r*[35] = 0.555, *p* < .001) and trended towards predicting faster rapid naming (*r*[35] = -0.301, *p* = .070). Crucially, in the 3-y-olds the model predicted rapid naming (*r*[18] = -0.692, *p* = .001), meaning that applying the model derived in Experiment 1 generalizes both to a new cohort and a new preliteracy skill; however, it did not predict 3-y-old’s memory for spoken sentences (*r*[18] = 0.034, *p* = 0.888). Scatterplots for these correlations are shown in S3 Fig.

### Neural Coding of Consonants in Noise Predicts Future Performance on Literacy Tests (Experiment 3)

**Fig 3:**
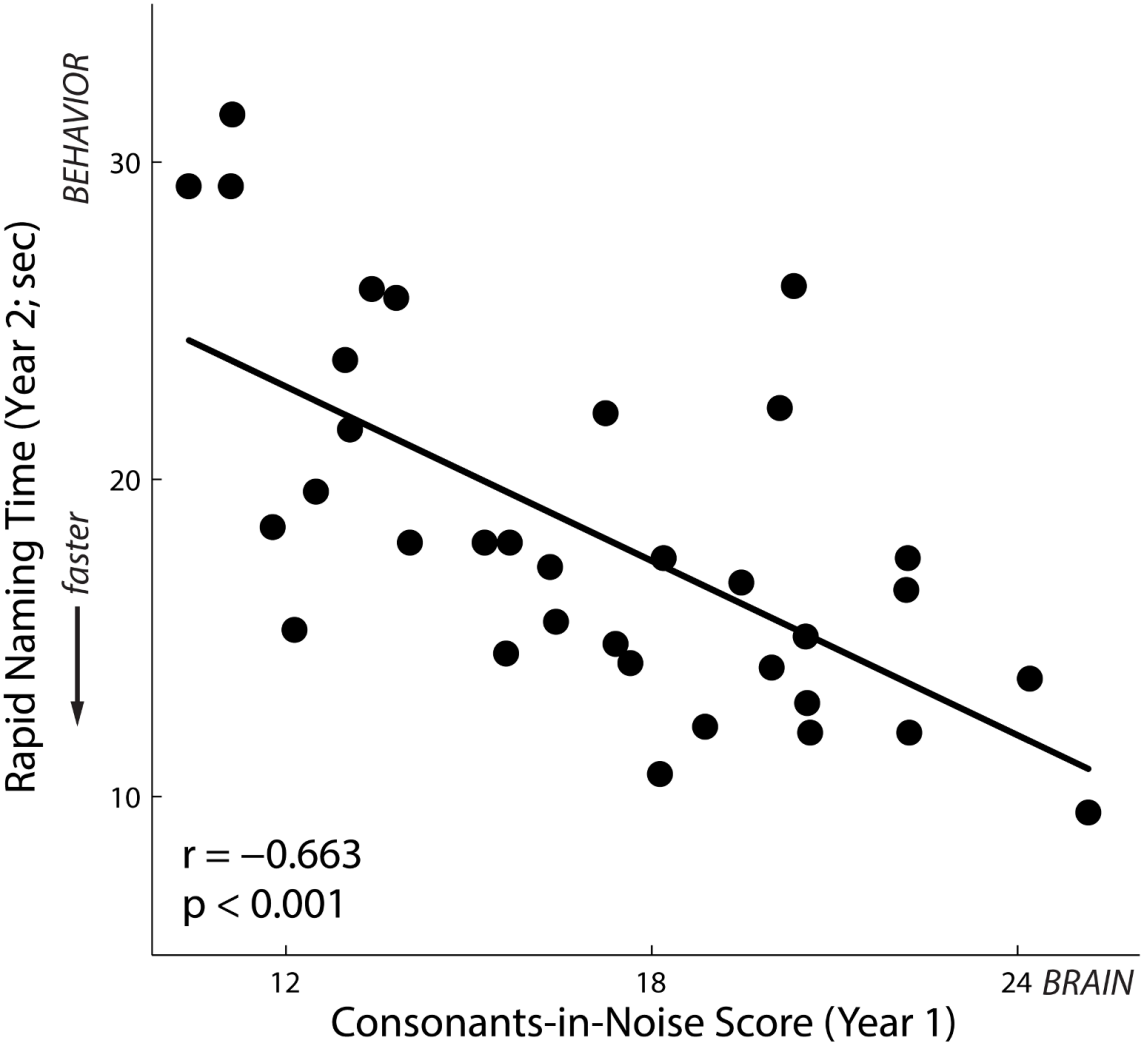
In preschoolers (*n* = 34), model predictions of phonological processing in Year 1 (based on auditory neurophysiology) predict rapid automatized naming time in Year 2, with higher predicted scores correlating with faster naming times for objects and colors (*r* = -.663, *p* < .001). Please refer to the S1 Data for data underlying this figure.

A subset of children from Experiments 1 and 2 returned one year later for a behavioral test battery (*n* = 34, 18 female). We took the “consonants-in-noise score” derived from the model in Experiment 1 and explored relations between the model’s predictions and performance on a variety of literacy tests one year after neurophysiological assessment. Year 1 neurophysiological testing predicted future performance on the same test of phonological processing—including in children too young to take this test in Year 1 (*r*[32] = 0.543, *p* = .001). These predictions generalized to future performance on a second test of phonological processing (*r*[32] = 0.575, *p* < .001) and predicted future performance on the same test of rapid automatized naming (*r*[32] = -0.663, *p* < .001; see Fig 3) and the same test of memory for spoken sentences (*r*[32] = 0.458, *p* = 0.006).

In the second year we also administered tests to evaluate early literacy. Neurophysiological model predictions at Year 1 predicted future performance on sight word reading (*r*[32] = 0.476, *p* = .004), spelling (*r*[32] = 0.415, *p* = .015), and a composite reading score (*r*[32] = 0.425, *p* = .012; see S4 Fig). Thus, the neural coding of consonants in noise predicts future reading achievement on standardized tests, in addition to multiple substrate literacy skills.

### Neural Coding of Consonants in Noise Predicts Reading and Diagnostic Category in Older Children (Experiment 4)

In Experiments 1–3, we established an auditory-neurophysiological biomarker for pre-reading skills in preschoolers. We applied the regression model from Experiment 1 to a cohort of older children (*n* = 55, 22 female, ages 8–14 y, M = 10.82, SD = 1.7) in whom we collected identical auditory-neurophysiological responses (previously described in [15]). This allowed us to ask whether the “consonants-in-noise score” derived in the 4-y-old children generalizes to a different age group, and effectively predicts how these children would have performed on the preschool tests of phonological processing, given their precision of coding consonants in noise. In school-aged children, the neural coding of consonants in noise predicted concurrent reading competence (*r*[53] = 0.430, *p* = .001) and performance on a range of literacy tests including sight word reading (*r*[53] = 0.408, *p* = .002), non-word reading (*r*[53] = 0.329, *p* = .014), spelling (*r*[53] = 0.327, *p* = .015), oral reading efficiency (*r*[53] = 0.319, *p* = .018), and phonological processing (*r*[53] = 0.474, *p* < .001; see S5 Fig).

A subset of these children had been externally diagnosed with a learning disability (LD; *n* = 26); the diagnostic groups differed on their predicted scores (*F*[1,53] = 14.541, p < .001) and model predictions reliably classified children into diagnostic categories (discriminant function analysis: 69.1% of cases correctly classified, *λ* = 0.785, *χ* ^2^ = 12.728, *p* < .001). A receiver operating characteristic (ROC) analysis (see S6 Fig) revealed that the model score excelled in identifying if a child was not in the reading-impaired group (area under the curve [AUC] = 0.756; 95% confidence interval [CI], 0.627, 0.885; *p* = .001). From a clinical standpoint, this suggests that our consonants-in-noise approach may be most effective in “clearing” children as unlikely to develop an LD, thereby motivating thorough follow-up in the remaining children.

## Discussion

A well-acknowledged gap in our understanding of the biology of reading is what biological constraints are instantiated in the nervous system prior to reading instruction. Ours is, to our knowledge, one of the first studies to demonstrate a physiological–phonological coupling in an age group sufficiently young to preclude confounds from prolonged and formal reading experience. In this respect our findings are consistent with the view that phonological processing is a necessary foundational skill for reading development [8,24]. By establishing brain–behavior links in pre-readers that are carried through to school-aged children, our findings suggest a causal, and not simply correlative, role for auditory processing in learning to read. Because the integrity of neural speech processing is linked to phonological awareness (to date, perhaps the best conventional predictor of a child’s eventual reading achievement [37]) we suggest that the neurophysiological markers we report here provide a biological looking glass into a child’s future literacy.

Indeed, we show that our model predicts performance on reading readiness tests one year after neurophysiological assessment. In many cases, behavioral tests were not standardized for children as young as we could evaluate neurophysiologically. Moreover, we show that, in school-aged children, our model predicts literacy and diagnostic category. Thus, in cases of learning disabilities, this biomarker may represent pre-existing problems with forming sound-to-meaning and/or letter-to-sound connections that cause problems for children when they begin reading instruction, an interpretation in line with converging biological evidence [27,38].

The correlations between neural coding and literacy skills were somewhat weaker in school-aged children than in pre-readers; this is consistent with the view that reading subskills mature as a function of reading experience, and that phonological processing may not play as strong a role in literacy competence for older children as it does during the early stages of reading acquisition [39,40]. Moreover, older children may have developed compensatory strategies that reduce the influence of phonological processing on reading that contributed to this developmental uncoupling. Nevertheless, it is noteworthy that there was a consistent brain–behavior relationship observed from ages 3–14. Taken together with the breadth of relationships observed across preliteracy skills (i.e., both phonological processing and rapid naming), the neural coding of consonants in noise may reflect a child’s core literacy potential.

Pharmacological studies have suggested that the neurophysiological metrics in our model rely on inhibitory neurotransmitter function; a loss of inhibitory receptors and/or an excitatory-inhibitory misbalance in auditory midbrain is linked directly to a decrease in the synchronous neural firing necessary to encode dynamic speech features such as consonants [41], especially in adverse listening conditions. In fact, this subcortical neural synchrony is necessary for auditory processing in noise [33]. We therefore speculate that the biomarker revealed here may rely on the emergence of robust inhibitory function. By measuring suprathreshold responses to consonants in noise, we may have sufficiently taxed the developing auditory brain to reveal systematic individual differences in inhibitory processing. Individual differences in these functions may create challenges when children are trying to map sounds to meaning in noisy environments, potentially interfering with the development of the range of preliteracy skills correlated to auditory-neurophysiological responses here.

Our view is that this subcortical neural synchrony emerges and is honed through a distributed, but integrated, auditory circuit. With respect to reading, auditory cortical processing is thought to bootstrap the development of fluent speech processing; eventually, children begin to associate orthographic representations with mental representations of phonemes [8,10,17]. A breakdown in this integrative process may cause a reduction in corticofugal input in auditory midbrain (our biomarker’s putative generator), especially for acoustic transients in challenging listening environments (i.e., consonants in noise). This faulty processing may be due to poor phaselocking [10], abnormal thalamic and cortical cytoarchitectonics [38,40,42–44], and/or sluggish attentional resources [45]. Should a child fail to learn what to pay attention to in everyday listening environments, and in turn fail to allocate appropriate attentional resources to these relevant speech cues, he or she may struggle to build robust phonemic representations. This sound-meaning disjunction may disrupt the course of auditory learning, leading to suboptimal input from corticocollicular fibers and cascading to a decrease in inhibitory function at the cost of synchronous firing by midbrain nuclei [41]. In turn, without the development of refined neural coding, maladaptive compensatory mechanisms may develop that stanch the development of automaticity in reading and auditory processing in a feed-forward, feed-back loop. This view is consistent with evidence that substrate reading skills (such as phonological processing) and sensory processing develop as a function of reading experience [25,46]. Of course, this is speculative; we must infer midbrain function from far-field electrophysiological recordings. Nevertheless, it is intriguing to contemplate the role of inhibitory neurotransmission, and neurochemical mechanisms more broadly, with respect to language development [47].

Conventional tests of early literacy can be unreliable in children this young, and to our knowledge, standardized tests of phonological processing are not available for children younger than age 4. Moreover, children who perform poorly on these tests have the least reliable scores because the fewest items are administered, thereby increasing potential bias from a false positive. Given the comorbidity between reading disorders and other LDs, compliance with paper-and-pencil tests may be even lower in the children who stand at the highest risk for a disability and are the most important cases to screen. When these evaluations are available, they are most reliable in identifying a child at risk for a LD, rather than systematically predicting a child’s position along a continuum of literacy achievement. The same may be said for previously established neurophysiological predictors of a child’s diagnosis [28,48]. We do not make these claims to denigrate the contributions of other research groups, or the obvious fact that, in many cases, simple paper-and-pencil tests and surveys can be effective in evaluating a child’s risk for a learning problem. Rather, our view is that by establishing these brain–behavior links in preschool children, our findings can pave the way for auditory-neurophysiological assessment in even younger children, in addition to children who are difficult to test using conventional means.

Our approach was to combine multiple measures of neural coding to see how they collectively predict preliteracy skills; although all came from the same neurophysiological recording, each provided unique information and they were only modestly intercorrelated (average *r* = 0.318). Future work should focus on the similarities and differences between these measures. On the one hand, we provide evidence that in combination they predict several preliteracy skills and diagnostic category. On the other hand, reading impairment can arise for a number of reasons, which may have distinct pathophysiologies [49]. An intriguing possibility is that these different aspects of neural coding are uniquely linked to different etiologies of reading impairment and/or substrate reading skills.

These children will continue to be followed longitudinally to better understand the role this neural coding in noise plays in language development. From a theoretical perspective, we hope to elucidate how consonant processing in noise guides the development of literacy skills, especially in interactions with the distributed-but-integrated neural networks involved in auditory learning. Children with particularly poor processing of speech in noise may face challenges during critical auditory mapping experiences [50], inhibiting the development of precise neural coding. It would appear that we have established a neural correlate of preliteracy that is carried through to school age, precedes explicit reading instruction, and predicts both a child’s performance along a continuum of literacy and diagnostic category; it will be necessary, however, to replicate these findings in a larger sample. Pragmatically, our findings have the potential to facilitate both early diagnosis and interventions to improve literacy before a child begins explicit instruction. Efforts to promote literacy during early childhood can be tremendously effective, and our hope is that these results open a new avenue of early identification to provide children access to these crucial interventions.

## Materials and Methods

The Institutional Review Board of Northwestern University approved all study procedures in accordance with the Declaration of Helsinki. Parents or legal guardians provided written informed consent and children provided verbal assent to participate. Subjects were remunerated for their participation.

### Subjects

Children were recruited from the Chicago area. No child had a history of a neurologic condition, diagnosis of autism spectrum disorder, or second language experience (all were native English speakers). In all cases children had normal auditory brainstem responses (elicited by a 100 μs square-wave click presented at 80 dB SPL to the right ear at 31.3 Hz; Navigator Pro, Bio-Logic Systems, Mundelein, IL, United States).

Preschoolers (Experiments 1–3) passed a screening of peripheral auditory function (normal otoscopy, Type A tympanograms, distortion product otoacoustic emissions ≥ 6 dB SPL above the noise floor from 0.5–4 kHz). School-aged children (Experiment 4) passed an audiometric screening (air-conduction thresholds ≤15 dB HL at octaves from 0.250–8 kHz bilaterally with no evidence of a conductive hearing loss and distortion product otoacoustic emissions ≥6 dB SPL above the noise floor from 0.5–4 kHz).

### Stimulus

Frequency-following responses were elicited to a 170 ms [da] stimulus. The [da] is a voiced (5 ms voice onset time) six-formant stop consonant constructed in a Klatt-based synthesizer at 20 kHz. Following the initial stop burst is a 50 ms consonant transition (/d/ to /a/) during which the lower three formants shift in frequency (F_1_ 400–720 Hz, F_2_ 1,700–1,240 Hz, F_3_ 2,580–2,500 Hz); these formants are steady for the subsequent 120 ms vowel (/a/). The fundamental frequency and upper three formants are steady throughout the stimulus (F_0_ 100 Hz, F_4_ 3,300 Hz, F_5_ 3,750 Hz, F_6_ 4,900 Hz).

The stimulus was presented against a six-talker babble track at a +10 SNR. The babble track consists of six talkers (three female) speaking semantically-anomalous English sentences. The 4,000 ms babble track is looped continuously such that there is no phase synchrony between the onsets of the [da] and noise.

The [da] and noise were mixed into a single channel that was presented to the right ear at 80 dB SPL in alternating polarities through electromagnetically-shielded insert earphones (ER-3A, Etymotic Research, Elk Grove Village, IL, US).

#### Experiments 1–3

Stimulus presentation was controlled by E-Prime 2.0 (Psychology Software Tools, Inc., Sharpsburg, PA, US) with an 81 ms interstimulus interval. There were 4,200 sweeps of the stimulus presented.

#### Experiment 4

Stimulus presentation was controlled by Neuroscan Stim 2 (Compumedics, Inc., Charlotte, NC, US) with a 61 ms interstimulus interval. There were 6,300 sweeps of the stimulus presented.

### Recording

Children sat in an electrically shielded and sound-attenuated booth (IAC Acoustics, Bronx, NY, US) and sat in a comfortable chair for recording while watching a film of their choice. The left ear remained unoccluded so the children could hear the movie soundtrack (~40 dB SPL).

#### Experiments 1–3

FFRs were recorded with a BioSEMI Active2 recording system with an auditory brainstem response (ABR) module. Active electrodes were placed at Cz and each ear, with CMS/DRL placed on the forehead, 1 cm on either side of Fpz (all offsets <50 mV). Only ipsilaterally referenced (Cz-A2) responses are considered in analyses; however, they likely reflect activity bilaterally [51]. Responses were digitized at 16.384 kHz with online filters set from 100–3,000 Hz (20 dB/decade roll-off) in the BioSEMI ActiABR module for LabView 2.0 (National Instruments, Austin, TX, US). To facilitate comparisons with Experiment 4, responses were amplified offline in the frequency domain using custom software in MATLAB (The Mathworks, Inc., Natick, MA, US). Responses were amplified 20 dB per decade for 3 decades below 100 Hz (0.1–100 Hz). Next responses were bandpass filtered to the frequency region of interest for the responses (70–2,000 Hz, Butterworth filter, 12 dB/octave roll-off, zero phase shift), epoched from -40–210 ms (stimulus onset at 0 ms), baselined, and artifact rejected (± 35 μV). Responses to alternating polarities were added; final averages comprised 4,000 sweeps.

#### Experiment 4

FFRs were recorded with a SynAmps2 system (Scan Acquire 4.3, Compumedics, Inc., Charlotte, NC, US). Electrodes were placed at Cz (active), A2 (reference), and Fpz (ground); all impedences were <5 kΩ. Responses were digitized at 20 kHz. Responses were filtered offline from (70–2,000 Hz, Butterworth filter, 12 dB/octave roll-off, zero phase shift), epoched from -40–190 ms (stimulus onset at 0 ms), baselined, artifact rejected (±35 μV). Responses to alternating polarities were added; final averages comprised 6,000 sweeps.

### Data Analyses

Our selection of metrics from the FFRs was motivated by previous investigations that have found links cross-sectionally between the timing, stability, and magnitude of responses to consonants and literacy skills. By using the same stimulus and recording scheme, we can apply uniform neurophysiological analyses across age groups. Please see [52] for technical guidance on FFR collection and analysis.

#### Neural timing

Positive-going deflections in the evoked responses (see Fig 1C) were identified by computer algorithm using local maximum detection (Scan Edit 4.3, Compumedics, Inc., Charlotte, NC, US). Peaks were labeled according to their expected latency (for example, a peak occurring 21–22 ms after stimulus onset would be called “Peak 21”). Peaks in response to the consonant transition are called Peaks 21, 31, 41, and 51. After they are identified by the algorithm, selections were adjusted manually using two sub-averages of a given response as a guide (see [15]). This procedure is performed blind to subject’s performance on behavioral tests.

#### Neural stability

To evaluate the trial-by-trial stability of the evoked responses, the filtered, epoched, and artifact-rejected responses were re-averaged using random selection 300 times to compute 300 pairs of sub-averages. Each sub-average comprised 50% of the trials in a recording (Experiments 1–3: 2,000 trials/sub-average; Experiment 4: 3,000 trials/sub-average). Each of the pairs of sub-averages was correlated and the mean correlation coefficient (Pearson’s *r*) was calculated over the response to the consonant (20–60 ms). The correlation coefficient was converted to a Fisher *z* coefficient for statistical purposes.

#### Representation of spectral features

A fast Fourier transformation (FFT) was applied on each response from 20–60 ms. The FFT was calculated with a 10 ms Hanning ramp and computed for harmonics at 400, 500, 600, and 700 Hz (40 Hz bins) to gauge the magnitude of responses to the first formant—a cue that contributes to phonemic identification. Spectral amplitudes across these four bins were averaged.

### Behavioral Test Battery

A series of standardized psychoeducational tests were administered. As much as possible, these tests were selected to provide overlap between experiments; however, we were constrained by the ages for which the tests were standardized and available. Please see Table 2 for a summary of each behavioral test broken down by experiment. The test battery included the Children’s Evaluation of Language Fundamentals-Preschool 2nd Edition (CELF-P2; Phonological Awareness and Recalling Sentences subtests; raw scores; Pearson, San Antonio, TX, US), the RAN (rapid automatized color and object naming; average naming time in seconds normalized on a log scale; PRO-ED, Inc., Austin, TX, US), the Comprehensive Test of Phonological Processing (CTOPP; 1st Edition for school-age children, 2nd Edition used for preschoolers; composite phonological awareness score used, standard score; Pearson, San Antonio, TX, US), the Woodcock-Johnson-III Tests of Achievement (WJ-III; Letter-Word Identification, Spelling, and Word Attack subtests and Basic Reading composite, standard scores; Riverside Publishing, Rolling Meadows, IL, US) and the Test of Word Reading Efficiency (TOWRE, standard scores; Pearson, San Antonio, TX, US). Non-verbal intelligence was evaluated in preschoolers with the Wechsler Preschool and Primary Scale of Intelligence-III (WPSSI-III, Object Assembly in 3-y-olds and Matrix Reasoning in 4-y-olds; scale scores; Pearson, San Antonio, TX, US) and in school-age children with the Wechsler Abbreviated Scale of Intelligence (WASI, Matrix Reasoning and Block Design subtests, standard scores; Pearson, San Antonio, TX, US).

**Table 2:** Behavioral test battery for each experiment.

	Test of …	Experiment			
		1	2	3	4
CELF-P2-Phonological Awareness	Phonological processing	X		X	
CELF-P2 Recalling Sentences	Auditory memory and grammar		X	X	
RAN	Rapid automatized naming		X	X	
CTOPP-Phonological Awareness^a^	Phonological processing			X	X
WJIII-Letter Word ID	Sight word reading			X	X
WJIII-Word Attack	Non-word reading				X
WJIII-Spelling	Spelling			X	X
WJIII-Basic Reading	Reading achievement			X	X
TOWRE	Oral reading efficiency				X
WPSSI-III	Non-verbal IQ	X	X	X	
WASI	Non-verbal IQ				X

^a^In Experiment 3, the CTOPP-2 test was used, whereas the CTOPP was used in Experiment 4.

### Statistical Modeling

Hierarchical regression was used to predict phonological processing from neurophysiological recordings. The first step comprised demographic factors (age, sex, and non-verbal intelligence) and the second step comprised neurophysiological factors; thus, the model estimates what percentage of variance in phonological processing neural coding accounts for above and beyond demographics.

The model constructed in Experiment 1 was applied to all subjects; on its first step there was a trend for demographics to significantly predict phonological processing (*R* ^2^ = 0.183, *F*[3,37] = 2.547, *p* = 0.072). In preliminary modeling, independent two-step regressions were run for each neurophysiological metric. In all cases, the neurophysiological metrics in isolation improved model fit (neural timing: Δ*R* ^2^ = 0.245, *F*[4,29] = 3.166, *p* = 0.028; representation of first formant: Δ*R* ^2^ = 0.254, *F*[4,29] = 3.340, *p* = 0.023; neural stability: Δ*R* ^2^ = 0.142, *F*[1,32] = 6.166, *p* = 0.013). These regression results are presented in S2 Table as Steps 2A, 2B, and 2C, respectively. Despite these metrics coming from a single recording, the overall model had acceptable levels of collinearity (tolerance ranged from 0.383–0.994), indicating that the model was not skewed by intercorrelations between predictors. All variables met the assumptions of the general linear model (i.e., normal distribution and heterogeneity of variance) and *p*-values reflect two-tailed tests.

## Supporting Information

S1 Data The dataset with original data for each of the figures in the manuscript and supporting information. (XLSX)

S1 Fig Correlations between neural coding measures and phonological processing used in Experiment 1. The neural timing measures (latencies of Peaks 21, 31, 41, and 51) are labeled in blue. The spectral measures (amplitudes at harmonics H_4_, H_5_, H_6_, and H_7_) are labeled in red. Neural stability (intertrial correlation in response to the consonant) is labeled in green, and phonological processing (CELF P-2 Phonological Awareness) is labeled in gray. Scatterplots on the lower left side of the figure shows the relations between these measures (z-transformed so that they are all on the same scale). The upper right side of the figure reports the zero-order correlation (larger font) and the partial correlation controlling for demographic factors (smaller gray font); italicized coefficients represent statistically-significant correlations (*p* < .05).
(TIF)

S2 Fig Results of the cross-validation analysis from Experiment 1. (A) Twenty subjects were chosen at random; the model was re-fit to them, and reliably predicted their phonological processing. (B) When this model is applied to the 17 remaining subjects, the neural coding of consonants in noise still predicts their phonological processing.
(TIF)

S3 Fig Scatterplots showing the relations between predictions from the consonants in noise model and performance on additional tests of preliteracy, with the correlations across age groups. The 4-y-olds from Experiment 1 are represented by dots and the 3-y-olds who were added in Experiment 2 by triangles. (A) Neural coding of consonants in noise predicts rapid naming. (B) Neural coding of consonants in noise predicts memory for sentences. (C) The correlation between rapid naming and memory for sentences is illustrated.
(TIF)

S4 Fig Correlations between the “consonants-in-noise” neural coding score in Year 1 (shaded in gray), and performance on tests of literacy subskills and tests of reading achievement in Year 2. Neural coding of consonants in noise predicts a range of skills, and in the case of rapid automatized naming provides a stronger prediction of future performance than the behavioral tests of phonological processing used to derive the model. Scatterplots on the lower left side of the figure show the relations between these measures (z-transformed so that they are all on the same scale). The upper right side of the figure reports the zero-order correlation; italicized coefficients represent statistically-significant correlations (*p* < .05).
(TIF)

S5 Fig Correlations between the neural coding “consonants-in-noise” score and measures of literacy achievement in the children from Experiment 4. The neural coding model (based on Experiment 1) predicts performance on a variety of literacy tests. Scatterplots on the lower left side of the figure show the relations between these measures (z-transformed so that they are all on the same scale). The upper right side of the figure reports the zero-order correlation; all correlations are statistically significant (*p* < 0.05).
(TIF)

S6 Fig Results of the classification analysis for diagnostic group from Experiment 4. The ROC curve classifying children into diagnostic groups is illustrated. The model is most reliable in “clearing” children as typically developing (i.e., here sensitivity refers to the likelihood of correctly identifying a child as in the control group).
(TIF)

S1 Table Regression table of the results of the cross-validation analysis from Experiment 1. The analysis is described in S1 Text. ^a^Dummy-coded, males = 0, females = 1. ~*p* = 0.63, *p ≤ 0.05.
(DOCX)

S2 Table Results of preliminary modeling that led to the regression model reported in Experiment 1. Neural timing (Step 2A), representation of the first formant (Step 2B), and neural stability (Step 2C) each predict phonological processing in isolation, over and above demographic factors (Step 1). ^a^Dummy-coded, males = 0, females = 1. ~*p* < 0.10, *p < 0.05, ** *p* < 0.01.
(DOCX)

S1 Text The cross-validation analysis from Experiment 1 is described. The cross-validation tested the generalizability of the regression model.
(DOCX)

## Abbreviations

ABR: auditory brainstem response
AUC: area under the curve
cABR: auditory brainstem response to complex sounds
CI: confidence interval
FFR: frequency-following response
LD: learning disability
M: mean
ROC: receiver operating characteristic
SD: standard deviation
SNR: signal-to-noise ratio
SPL: sound pressure level
