# Supplementary figures and images for: Auditory Processing in Noise: A Preschool Biomarker for Literacy
Source: PLoS Biol. 2015 Jul 14;13(7):e1002196. doi: 10.1371/journal.pbio.1002196 (PMC4501760; doi:10.1371/journal.pbio.1002196)

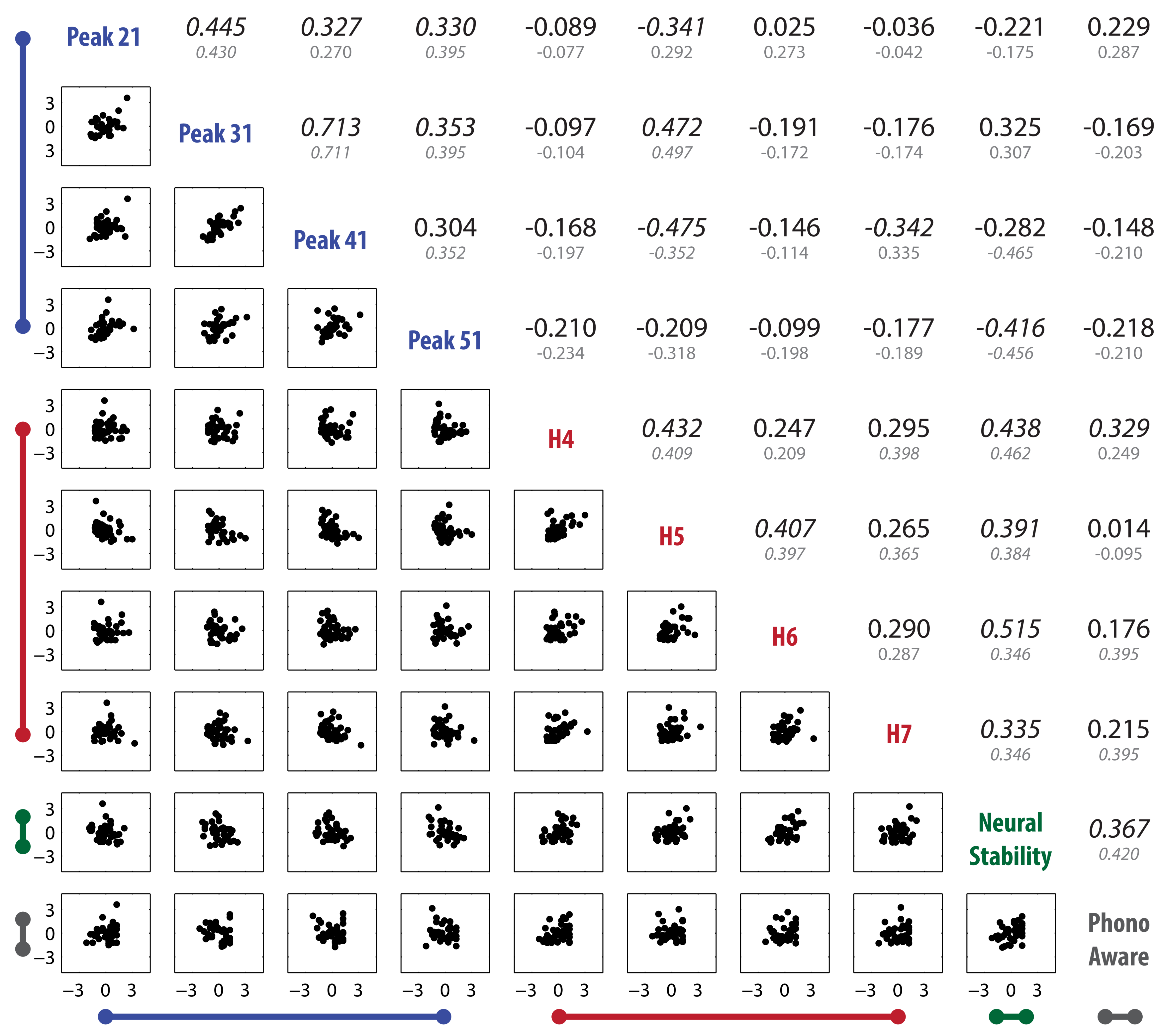

Supplement: S1 Fig — The neural timing measures (latencies of Peaks 21, 31, 41, and 51) are labeled in blue. The spectral measures (amplitudes at harmonics H4, H5, H6, and H7) are labeled in red. Neural stability (intertrial correlation in response to the consonant) is labeled in green, and phonological processing (CELF P-2 Phonological Awareness) is labeled in gray. Scatterplots on the lower left side of the figure shows the relations between these measures (z-transformed so that they are all on the same scale). The upper right side of the figure reports the zero-order correlation (larger font) and the partial correlation controlling for demographic factors (smaller gray font); italicized coefficients represent statistically-significant correlations (p < .05). (TIF) [file pbio.1002196.s002.tif]

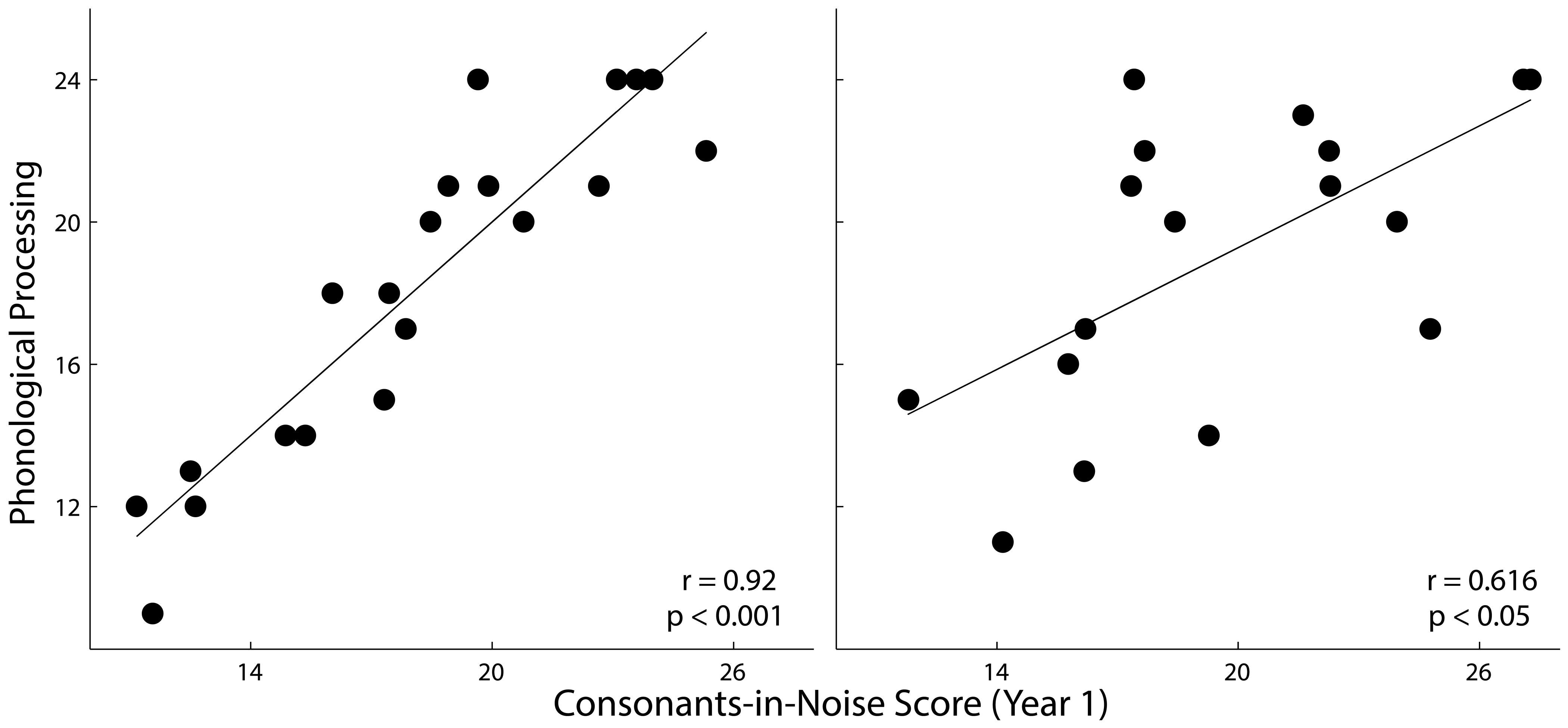

Supplement: S2 Fig — (A) Twenty subjects were chosen at random; the model was re-fit to them, and reliably predicted their phonological processing. (B) When this model is applied to the 17 remaining subjects, the neural coding of consonants in noise still predicts their phonological processing. (TIF) [file pbio.1002196.s003.tif]

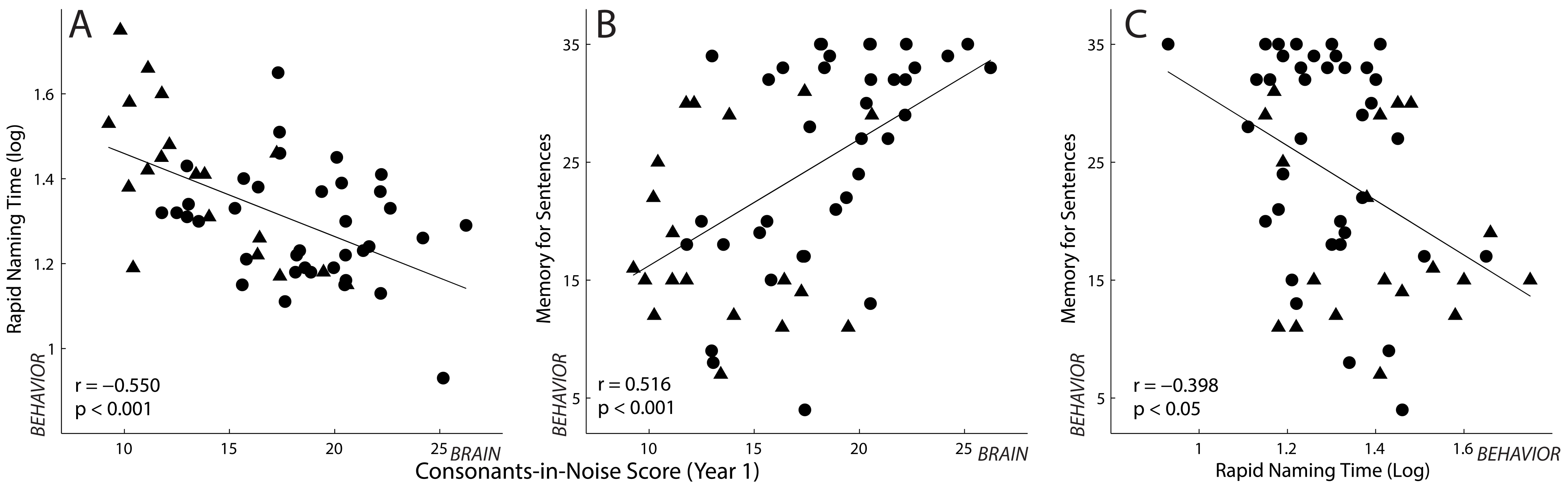

Supplement: S3 Fig — The 4-y-olds from Experiment 1 are represented by dots and the 3-y-olds who were added in Experiment 2 by triangles. (A) Neural coding of consonants in noise predicts rapid naming. (B) Neural coding of consonants in noise predicts memory for sentences. (C) The correlation between rapid naming and memory for sentences is illustrated. (TIF) [file pbio.1002196.s004.tif]

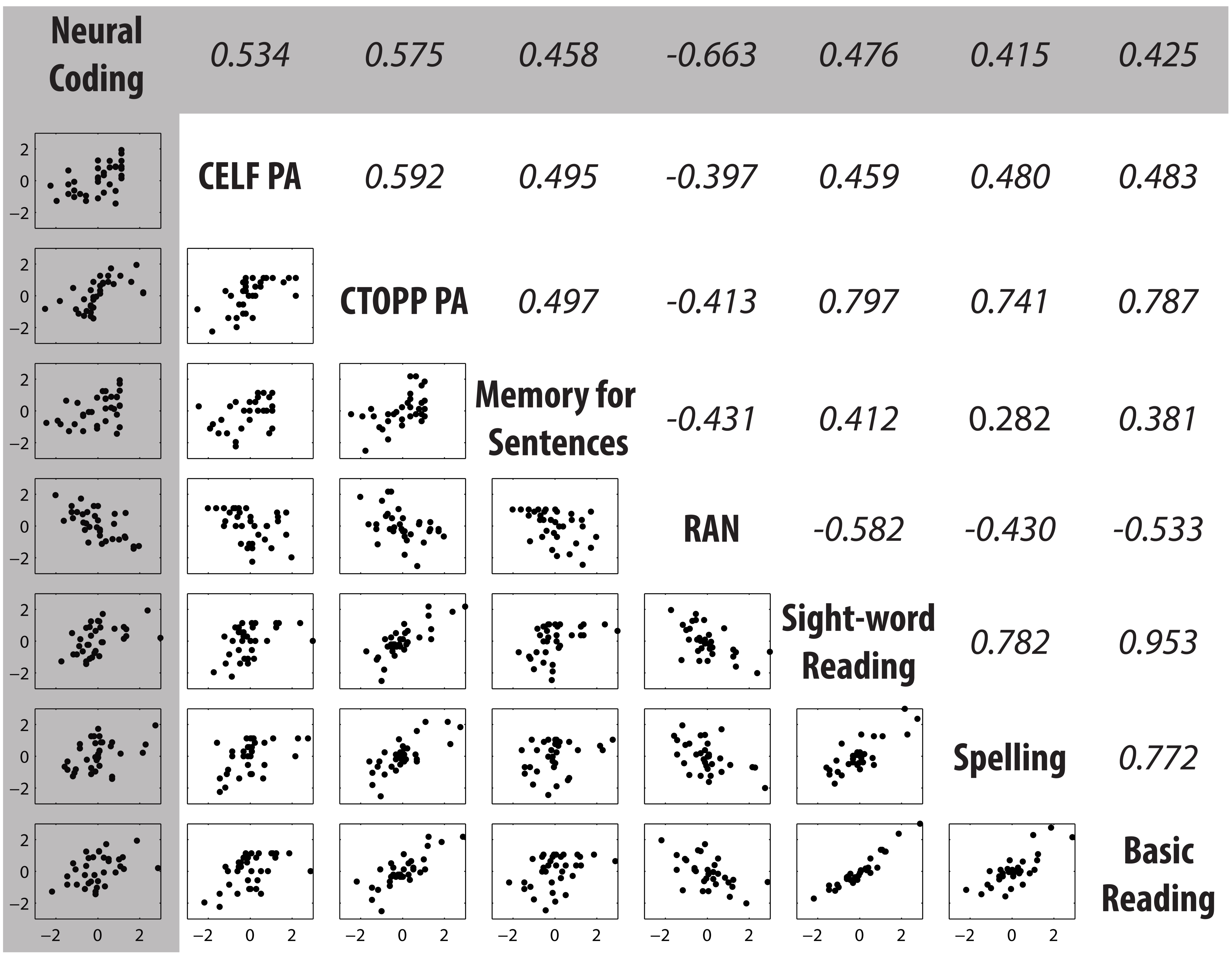

Supplement: S4 Fig — Neural coding of consonants in noise predicts a range of skills, and in the case of rapid automatized naming provides a stronger prediction of future performance than the behavioral tests of phonological processing used to derive the model. Scatterplots on the lower left side of the figure show the relations between these measures (z-transformed so that they are all on the same scale). The upper right side of the figure reports the zero-order correlation; italicized coefficients represent statistically-significant correlations (p < .05). (TIF) [file pbio.1002196.s005.tif]

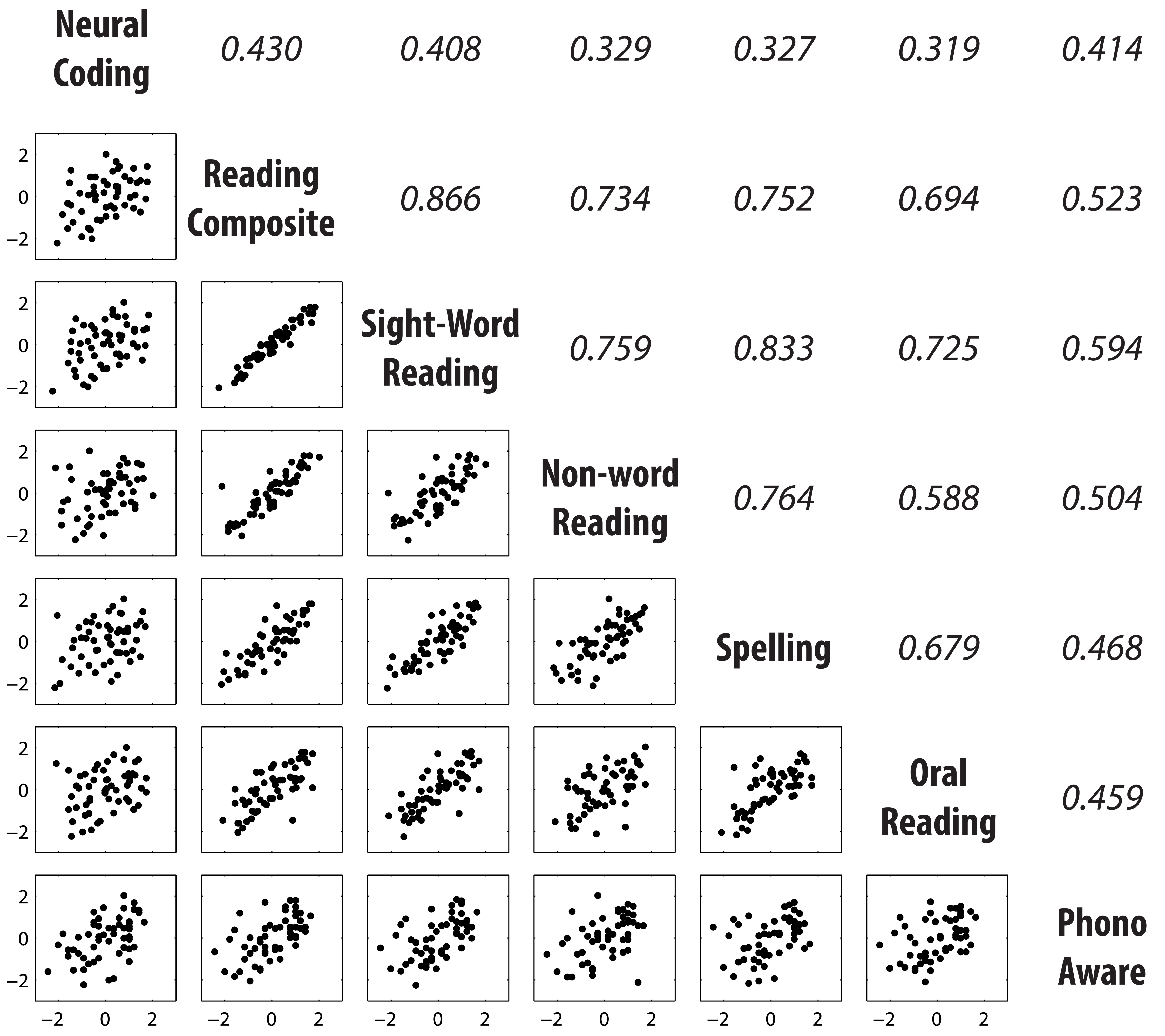

Supplement: S5 Fig — The neural coding model (based on Experiment 1) predicts performance on a variety of literacy tests. Scatterplots on the lower left side of the figure show the relations between these measures (z-transformed so that they are all on the same scale). The upper right side of the figure reports the zero-order correlation; all correlations are statistically significant (p < 0.05). (TIF) [file pbio.1002196.s006.tif]

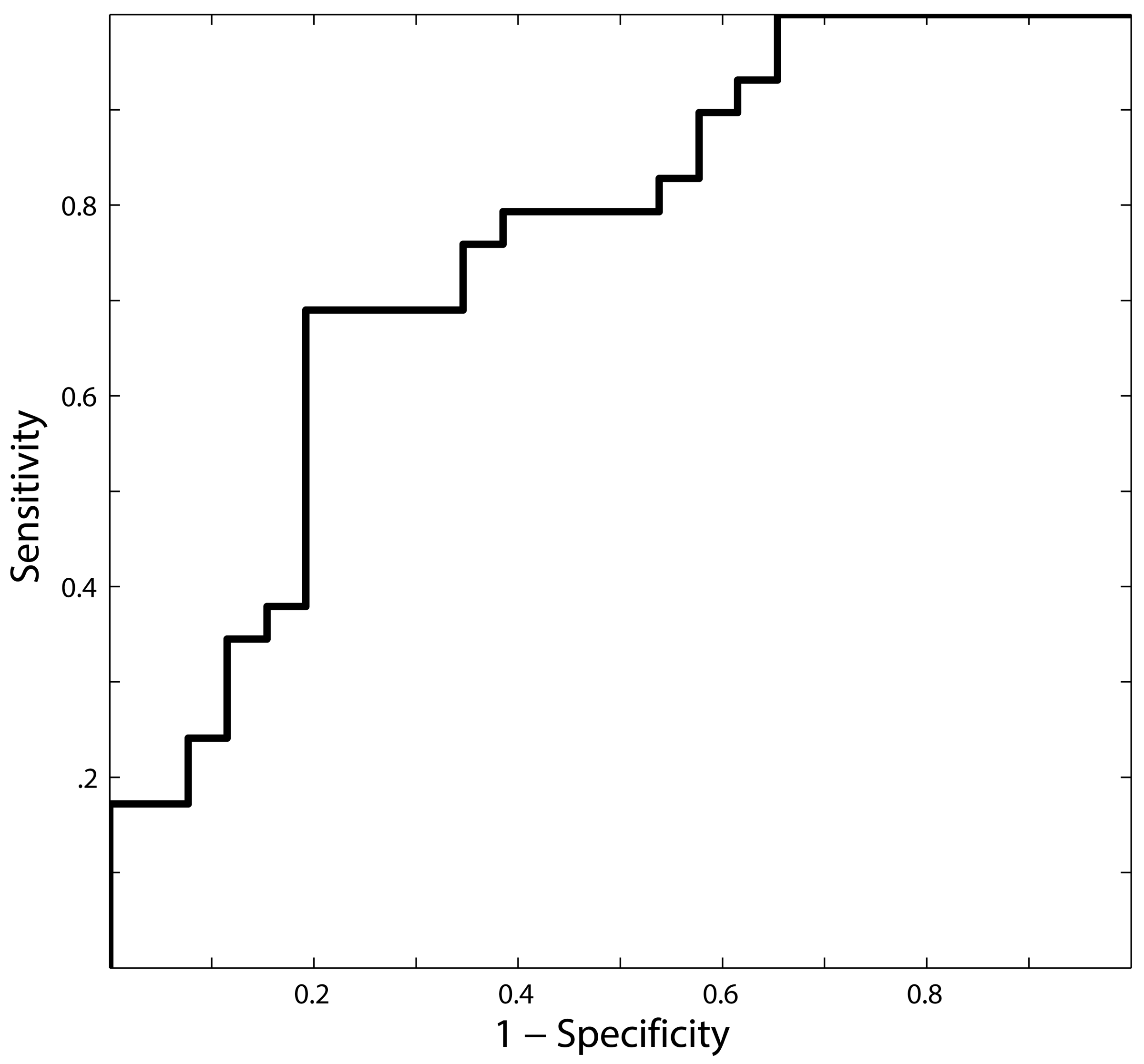

Supplement: S6 Fig — The ROC curve classifying children into diagnostic groups is illustrated. The model is most reliable in “clearing” children as typically developing (i.e., here sensitivity refers to the likelihood of correctly identifying a child as in the control group). (TIF) [file pbio.1002196.s007.tif]
