# Supplementary material for: Auditory Processing in Noise: A Preschool Biomarker for Literacy
Source: PLoS Biol. 2015 Jul 14;13(7):e1002196. doi: 10.1371/journal.pbio.1002196 (PMC4501760; doi:10.1371/journal.pbio.1002196)
Supplement: S1 Table — The analysis is described in S1 Text. aDummy-coded, males = 0, females = 1. ~p = 0.63, *p ≤ 0.05. (DOCX) [file pbio.1002196.s008.docx]

| **Predictor** | **Δ*R*^2^** | ***β*** |
| --- | --- | --- |
| Step 1 | 0.378* |  |
| Sex^a^ |  | -0.167 |
| Age |  | 0.527* |
| Non-verbal IQ |  | -0.351 |
|  |  |  |
| Step 2 | 0.470 |  |
| Sex |  | -0.247 |
| Age |  | 0.423 |
| Non-verbal IQ |  | 0.309 |
| *Neural timing* |  |  |
| Peak 21 |  | 0.583* |
| Peak 31 |  | -0.214 |
| Peak 41 |  | -0.108 |
| Peak 51 |  | -0.286 |
| *First formant* |  |  |
| H_4_ |  | 0.150 |
| H_5_ |  | -0.557 |
| H_6_ |  | 0.182 |
| H_7_ |  | 0.466 |
| *Neural stability* |  | 0.198 |
|  |  |  |
| **Total R^2^** | 0.848~ |  |

**Table S1.** Results of the cross-validation analysis from Experiment 1. ^a^Dummy-coded, males = 0, females = 1. ~*p* = 0.63, **p* ≤ 0.05
