# Supplementary material for: Auditory Processing in Noise: A Preschool Biomarker for Literacy
Source: PLoS Biol. 2015 Jul 14;13(7):e1002196. doi: 10.1371/journal.pbio.1002196 (PMC4501760; doi:10.1371/journal.pbio.1002196)
Supplement: S2 Table — Neural timing (Step 2A), representation of the first formant (Step 2B), and neural stability (Step 2C) each predict phonological processing in isolation, over and above demographic factors (Step 1). aDummy-coded, males = 0, females = 1. ~p < 0.10, *p < 0.05, ** p < 0.01. (DOCX) [file pbio.1002196.s009.docx]

| **Demographics** | **Δ*R*^2^** | ***Β*** |  | **Neural Timing** | **Δ*R*^2^** | ***β*** |  | **First Formant** | **Δ*R*^2^** | ***β*** |  | **Neural Stability** | **Δ*R*^2^** | ***β*** |
| --- | --- | --- | --- | --- | --- | --- | --- | --- | --- | --- | --- | --- | --- | --- |
| Step 1 | 0.196~ |  |  | Step 2A | 0.245* |  |  | Step 2B | 0.254* |  |  | Step 2C | 0.142* |  |
| Sex^a^ |  | -0.076 |  | Peak 21 |  | 0.512** |  | H_4_ |  | 0.173 |  | Stability |  | 0.386* |
| Age |  | 0.390* |  | Peak 31 |  | -0.242 |  | H_5_ |  | -0.409* |  |  |  |  |
| Non-verbal IQ |  | 0.114 |  | Peak 41 |  | -0.054 |  | H_6_ |  | 0.251 |  |  |  |  |
|  |  |  |  | Peak 51 |  | -0.312~ |  | H_7_ |  | 0.373* |  |  |  |  |
|  |  |  |  |  |  |  |  |  |  |  |  |  |  |  |
|  |  |  |  | **Total R^2^** | **0.440*** |  |  | **Total R^2^** | **0.449**** |  |  | **Total R^2^** | **0.337**** |  |

**Table S2.** Results of preliminary modeling that led to the regression model reported in Experiment 1. Neural timing (Step 2A), representation of the first formant (Step 2B), and neural stability (Step 2C) each predicts phonological processing in isolation, over and above demographic factors (Step 1).  ^a^Dummy-coded, males = 0, females = 1. ~*p* < 0.10, **p* < 0.05, ** *p* < 0.01.
