# Supplementary material for: Auditory Processing in Noise: A Preschool Biomarker for Literacy
Source: PLoS Biol. 2015 Jul 14;13(7):e1002196. doi: 10.1371/journal.pbio.1002196 (PMC4501760; doi:10.1371/journal.pbio.1002196)
Supplement: S1 Text — The cross-validation tested the generalizability of the regression model. (DOCX) [file pbio.1002196.s010.docx]

**Supporting Information - Results**

*Experiment 1 – Cross-validation analysis*

To assess the generalizability of the model predicted in Experiment 1, we performed a cross-validation. We randomly selected 20 of the 37 children in this experiment (12 female, mean age 55.2 months, SD 3.68) and reran the regression. Although this was somewhat underpowered, there was still an overall trend for neural coding to predict phonological processing more strongly than demographic factors (Δ*R*^2^ = 0.70, *F*(7,9) = 2.405, *p* = 0.130; total *R*^2^ = 0.848, *F*(7,12) = 3.252, *p* = 0.063; see Table S1). We then applied these regression weights to the 17 remaining children from this experiment and found that these predicted scores correlated to actual performance on the phonological processing test (*r*(15) = 0.616, *p* = .008; see Fig. S2).
